# Supplementary material for: Identification of personal factors that influence engagement in cardiac rehabilitation and interventions targeting personal factors: A scoping review protocol
Source: PLoS One. 2025 Jan 31;20(1):e0318265. doi: 10.1371/journal.pone.0318265 (PMC11785271; doi:10.1371/journal.pone.0318265)
Supplement: S2 File — (DOCX) [file pone.0318265.s002.docx]

**S2 file. Search strategy for PubMed**

| **Search number** | **Query** | **Results** |
| --- | --- | --- |
| 8 | (("Cardiac Rehabilitation"[Mesh]) OR (((((((Cardiac Rehabilitations[Title/Abstract]) OR (Rehabilitation, Cardiac[Title/Abstract])) OR (Rehabilitations, Cardiac[Title/Abstract])) OR (Cardiovascular Rehabilitation[Title/Abstract])) OR (Cardiovascular Rehabilitations[Title/Abstract])) OR (Rehabilitation, Cardiovascular[Title/Abstract])) OR (Rehabilitations, Cardiovascular[Title/Abstract]))) AND ((("Patient Participation"[Mesh]) OR (((((((((((((articipation, Patient[Title/Abstract]) OR (Patient Involvement[Title/Abstract])) OR (Involvement, Patient[Title/Abstract])) OR (Patient Participation Rates[Title/Abstract])) OR (Participation Rate, Patient[Title/Abstract])) OR (Participation Rates, Patient[Title/Abstract])) OR (Patient Participation Rate[Title/Abstract])) OR (Patient Engagement[Title/Abstract])) OR (Engagement, Patient[Title/Abstract])) OR (Patient Activation[Title/Abstract])) OR (Activation, Patient[Title/Abstract])) OR (Patient Empowerment[Title/Abstract])) OR (Empowerment, Patient[Title/Abstract]))) OR (("Enrolment"[tiab] OR "adherence"[tiab] OR "completion"[tiab] OR "participation"[tiab] OR "compliance"[tiab] OR "engag*"[tiab] OR "participat*"[tiab] OR "involv*"[tiab] OR "attend*"[tiab] OR "contin*"[tiab] OR "commit*"[tiab] OR "maint*"[tiab] OR "adhere*"[tiab] OR "uptake*"[tiab] OR "initiat*"[tiab] OR "referral*"[tiab] OR "self-referral*" [tiab] OR "recruit*"[tiab] OR "commenc*"[tiab] OR "inten*"[tiab] OR "enlist*"[tiab] OR "enrol*"[tiab] OR "sign up"[tiab] OR "take up"[tiab]))) | 18,943 |
| 7 | ("Cardiac Rehabilitation"[Mesh]) OR (((((((Cardiac Rehabilitations[Title/Abstract]) OR (Rehabilitation, Cardiac[Title/Abstract])) OR (Rehabilitations, Cardiac[Title/Abstract])) OR (Cardiovascular Rehabilitation[Title/Abstract])) OR (Cardiovascular Rehabilitations[Title/Abstract])) OR (Rehabilitation, Cardiovascular[Title/Abstract])) OR (Rehabilitations, Cardiovascular[Title/Abstract])) | 35,960 |
| 6 | ((((((Cardiac Rehabilitations[Title/Abstract]) OR (Rehabilitation, Cardiac[Title/Abstract])) OR (Rehabilitations, Cardiac[Title/Abstract])) OR (Cardiovascular Rehabilitation[Title/Abstract])) OR (Cardiovascular Rehabilitations[Title/Abstract])) OR (Rehabilitation, Cardiovascular[Title/Abstract])) OR (Rehabilitations, Cardiovascular[Title/Abstract]) | 35,523 |
| 5 | "Cardiac Rehabilitation"[Mesh] | 4,098 |
| 4 | (("Patient Participation"[Mesh]) OR (((((((((((((articipation, Patient[Title/Abstract]) OR (Patient Involvement[Title/Abstract])) OR (Involvement, Patient[Title/Abstract])) OR (Patient Participation Rates[Title/Abstract])) OR (Participation Rate, Patient[Title/Abstract])) OR (Participation Rates, Patient[Title/Abstract])) OR (Patient Participation Rate[Title/Abstract])) OR (Patient Engagement[Title/Abstract])) OR (Engagement, Patient[Title/Abstract])) OR (Patient Activation[Title/Abstract])) OR (Activation, Patient[Title/Abstract])) OR (Patient Empowerment[Title/Abstract])) OR (Empowerment, Patient[Title/Abstract]))) OR (("Enrolment"[tiab] OR "adherence"[tiab] OR "completion"[tiab] OR "participation"[tiab] OR "compliance"[tiab] OR "engag*"[tiab] OR "participat*"[tiab] OR "involv*"[tiab] OR "attend*"[tiab] OR "contin*"[tiab] OR "commit*"[tiab] OR "maint*"[tiab] OR "adhere*"[tiab] OR "uptake*"[tiab] OR "initiat*"[tiab] OR "referral*"[tiab] OR "self-referral*" [tiab] OR "recruit*"[tiab] OR "commenc*"[tiab] OR "inten*"[tiab] OR "enlist*"[tiab] OR "enrol*"[tiab] OR "sign up"[tiab] OR "take up"[tiab])) | 8,878,917 |
| 3 | ("Enrolment"[tiab] OR "adherence"[tiab] OR "completion"[tiab] OR "participation"[tiab] OR "compliance"[tiab] OR "engag*"[tiab] OR "participat*"[tiab] OR "involv*"[tiab] OR "attend*"[tiab] OR "contin*"[tiab] OR "commit*"[tiab] OR "maint*"[tiab] OR "adhere*"[tiab] OR "uptake*"[tiab] OR "initiat*"[tiab] OR "referral*"[tiab] OR "self-referral*" [tiab] OR "recruit*"[tiab] OR "commenc*"[tiab] OR "inten*"[tiab] OR "enlist*"[tiab] OR "enrol*"[tiab] OR "sign up"[tiab] OR "take up"[tiab]) | 8,865,525 |
| 2 | ((((((((((((articipation, Patient[Title/Abstract]) OR (Patient Involvement[Title/Abstract])) OR (Involvement, Patient[Title/Abstract])) OR (Patient Participation Rates[Title/Abstract])) OR (Participation Rate, Patient[Title/Abstract])) OR (Participation Rates, Patient[Title/Abstract])) OR (Patient Participation Rate[Title/Abstract])) OR (Patient Engagement[Title/Abstract])) OR (Engagement, Patient[Title/Abstract])) OR (Patient Activation[Title/Abstract])) OR (Activation, Patient[Title/Abstract])) OR (Patient Empowerment[Title/Abstract])) OR (Empowerment, Patient[Title/Abstract]) | 13,050 |
| 1 | "Patient Participation"[Mesh] | 30,126 |
