# Supplementary material for: Identification of personal factors that influence engagement in cardiac rehabilitation and interventions targeting personal factors: A scoping review protocol
Source: PLoS One. 2025 Jan 31;20(1):e0318265. doi: 10.1371/journal.pone.0318265 (PMC11785271; doi:10.1371/journal.pone.0318265)
Supplement: S3 File — (DOCX) [file pone.0318265.s003.docx]

**S3 file. PRISMA flow diagram**

**Identification of studies via databases**

Records removed *before screening*:

Duplicate records removed (n = )

Records marked as ineligible by automation tools (n = )

Records removed for other reasons (n = )

Records identified from*:

Databases (n = )

**Identification**

Records screened

(n = )

Records excluded**

(n = )

Reports sought for retrieval

(n = )

Reports not retrieved

(n = )

**Screening**

Reports assessed for eligibility

(n = )

Reports excluded:

Reason 1 (n = )

Reason 2 (n = )

Reason 3 (n = )

etc.

Studies included in scoping review

(n = )

**Included**

*Consider, if feasible to do so, reporting the number of records identified from each database or register searched (rather than the total number across all databases/registers).

**If automation tools were used, indicate how many records were excluded by a human and how many were excluded by automation tools.

*From:*  Page MJ, McKenzie JE, Bossuyt PM, Boutron I, Hoffmann TC, Mulrow CD, et al. The PRISMA 2020 statement: an updated guideline for reporting systematic reviews. BMJ 2021;372:n71. doi: 10.1136/bmj.n71
