# Supplementary material for: Identification of personal factors that influence engagement in cardiac rehabilitation and interventions targeting personal factors: A scoping review protocol
Source: PLoS One. 2025 Jan 31;20(1):e0318265. doi: 10.1371/journal.pone.0318265 (PMC11785271; doi:10.1371/journal.pone.0318265)
Supplement: S4 File — (DOCX) [file pone.0318265.s004.docx]

**S4 File. Data extraction instrument**

| **Study characteristics** |
| --- |
| Author(s) |
| Year |
| Country |
| Study design |
| Study setting |
| Study purpose |
| Theoretical framework |
| Stage of cardiac rehabilitation |
| Type of cardiac rehabilitation |
| Participants (age/sex and number) |
| **Personal factors of Cardiac Rehabilitation enrollment, adhenrence and complication** |
| Personal factors in emotion |
| Personal factors in cognition |
| Personal factors in behavior |
| **Intervention characteristics** |
| Purpose |
| Theoretical basis |
| Intervention components |
| Mode of delivery |
| Implementer |
| Duration |
| location |
| Targeted individual factors |
